# Immunological biomarkers predict HIV-1 viral rebound after treatment interruption

**DOI:** 10.1038/ncomms9495

**Published:** 2015-10-09

**Authors:** Jacob Hurst, Matthias Hoffmann, Matthew Pace, James P. Williams, John Thornhill, Elizabeth Hamlyn, Jodi Meyerowitz, Chris Willberg, Kersten K. Koelsch, Nicola Robinson, Helen Brown, Martin Fisher, Sabine Kinloch, David A. Cooper, Mauro Schechter, Giuseppe Tambussi, Sarah Fidler, Abdel Babiker, Jonathan Weber, Anthony D. Kelleher, Rodney E. Phillips, John Frater

**Affiliations:** 1 Nuffield Department of Clinical Medicine, Peter Medawar Building for Pathogen Research, John Radcliffe Hospital, Oxford OX1 3SY, UK; 2 Institute for Emerging Infections, The Oxford Martin School, Oxford OX1 3BD, UK; 3 Division of Medicine, Wright Fleming Institute, Imperial College, London W2 1PG, UK; 4 Caldecot Centre, King's College Hospital NHS Foundation Trust, London SE5 9RS, UK; 5 Oxford National Institute of Health Research Biomedical Research Centre, Oxford OX3 7LE, UK; 6 St Vincent's Centre for Applied Medical Research and The Kirby Institute, UNSW Australia, Sydney, New South Wales 2052, Australia; 7 Department of HIV and Sexual Health, Brighton and Sussex University Hospitals, Brighton BN2 5BE, UK; 8 Division of Infection and Immunity, University College London, London WC1E 6BT, UK; 9 Projeto Praça Onze, Hospital Escola São Francisco de Assis, Universidade Federal do Rio de Janeiro, Rio de Janeiro RJ 21941-901, Brazil; 10 Department of Infectious Diseases, Ospedale San Raffaele, Milan 20132, Italy; 11 MRC Clinical Trials Unit at UCL Institute of Clinical Trials & Methodology, London WC2B 6NH, UK

## Abstract

Treatment of HIV-1 infection with antiretroviral therapy (ART) in the weeks following transmission may induce a state of ‘post-treatment control' (PTC) in some patients, in whom viraemia remains undetectable when ART is stopped. Explaining PTC could help our understanding of the processes that maintain viral persistence. Here we show that immunological biomarkers can predict time to viral rebound after stopping ART by analysing data from a randomized study of primary HIV-1 infection incorporating a treatment interruption (TI) after 48 weeks of ART (the SPARTAC trial). T-cell exhaustion markers PD-1, Tim-3 and Lag-3 measured prior to ART strongly predict time to the return of viraemia. These data indicate that T-cell exhaustion markers may identify those latently infected cells with a higher proclivity to viral transcription. Our results may open new avenues for understanding the mechanisms underlying PTC, and eventually HIV-1 eradication.

## Abstract (web summary)

In some HIV-1-infected individuals, viraemia remains undetectable after antiretroviral treatment, but which of these patients will experience viral rebound is difficult to predict. Here the authors show that T cell exhaustion markers before treatment are predictive of shorter time to viral rebound.

## Graphical abstract

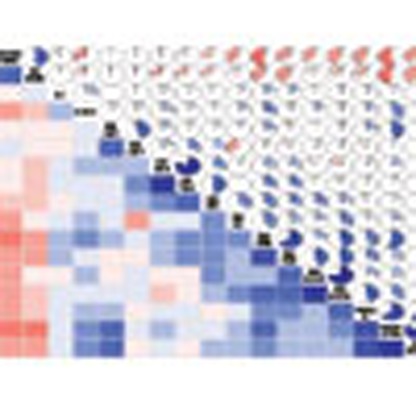

Antiretroviral therapy (ART) for the treatment of Human Immunodeficiency Virus Type 1 (HIV-1) infection has dramatically improved survival^1^,^2^,^3^, but is not a cure—a consequence of a reservoir of latently infected cells^4^. Primary HIV-1 infection (PHI)—the period within weeks to months of seroconversion—may provide insights into how this reservoir is activated and whether it can be suppressed, as stopping ART initiated in this early stage has in certain patients been associated with periods of aviraemic remission^5^—in some cases for over 10 years^6^. *Post hoc* analyses of other larger PHI cohorts support this phenomenon of sustained post-treatment control (PTC)^7^,^8^, as well as periods of transient aviraemic remission^5^.

The mechanisms that induce and maintain this remissive state of PTC remain unclear. There is debate over whether the timing of viral rebound after stopping ART is a random, stochastic process based on the spontaneous re-awakening of HIV-1 in latently infected T lymphocytes, or whether the host—for example, through HIV-1-specific or innate immunity—is the determining factor. In the best characterized cohort to date (VISCONTI), PTC was associated with low (but detectable) reservoirs, almost non-existent HIV-specific immune responses, an absence of protective HLA Class I alleles and highly symptomatic seroconversion illnesses^6^—a distinct phenotype from the more widely recognised examples of spontaneous viraemic control shown by ‘elite controllers'^9^. The SPARTAC trial^10^ was a randomized controlled trial (RCT) comparing ART for either 48 or 12 weeks relative to standard of care in adults with PHI. We reported prolonged periods of aviraemia post-treatment interruption (TI) in participants allocated to receive ART for 48 weeks—14% had undetectable plasma viral loads 1 year after stopping ART^5^. Total HIV-1 DNA level at baseline and week 48 was associated with shorter time to viral rebound^11^.

Here we report an analysis of immunological and virological factors from SPARTAC to explore those that either associate with HIV-1 DNA levels or independently predict viral rebound on stopping ART. We find that pre-therapy measures of T-cell exhaustion (PD-1, Tim-3 and Lag-3) were statistically significant predictors of virological rebound, suggesting that these markers should be considered in future algorithms used to detect PTC.

## Results

### SPARTAC trial participant characteristics

About 154 participants recruited to the SPARTAC trial^12^ were studied based on infection with subtype B HIV-1 and sample availability. SPARTAC incorporated a randomly allocated TI after 48 weeks of ART in a third of patients, providing a unique opportunity to study determinants of rebound. The demographics of the 154 participants are shown in Supplementary Table 1. All 154 patients were sampled for HIV-1 DNA levels at enrolment at the pre-therapy baseline (week 0), a median of 74 days from the estimated date of HIV-1 seroconversion. Of these (due to sample limitations), 78 were also assayed for immunological markers of T-cell exhaustion and activation (Supplementary Table 1). Participants who were randomized to receive 48 weeks of ART, and for whom samples were available at the point of stopping ART, (*n*=47; Supplementary Table 1) were studied in analyses of TI.

### Selection of biomarkers

We devised a panel of 18 biomarkers to measure parameters of host immunity and markers of the HIV-1 reservoir (Table 1). We wished to determine associations with levels of HIV-1 DNA levels and assess whether the biomarkers might also independently predict rebound at TI. At baseline, biomarker assays were carried out on up to 154 patients (not all assays were conducted on all patients—Table 1 for breakdown). At the point of TI after 48 weeks of ART, 47 participants were available for analysis. Again, and due to sample availability, not all biomarkers were tested on all patients (Table 1).

### T-cell immunity correlates with HIV-1 DNA at seroconversion

**Figure 1:**
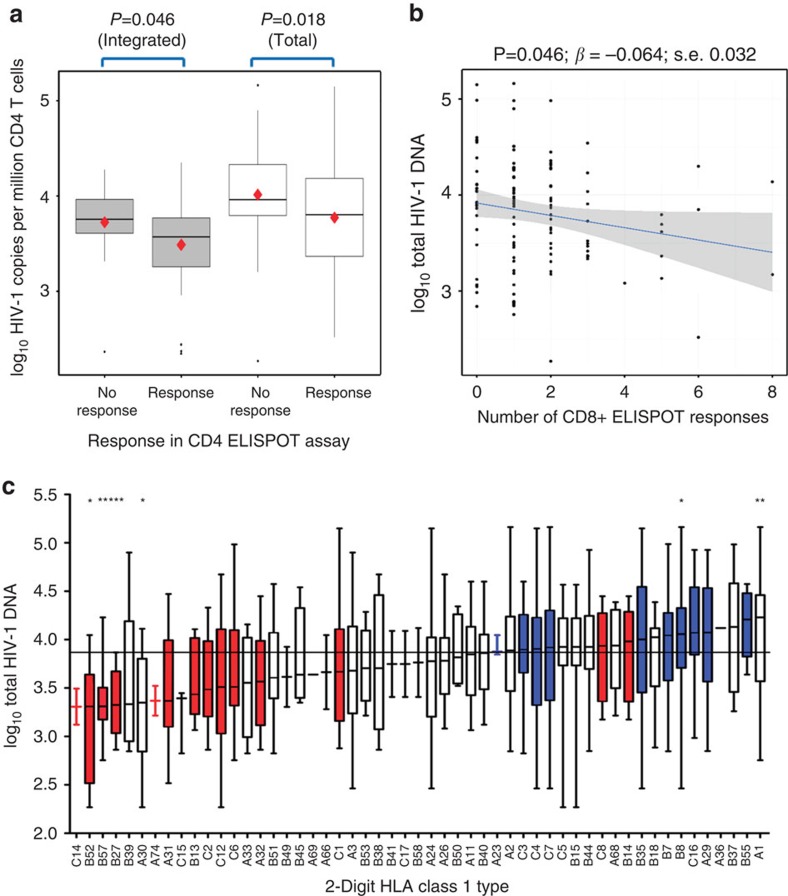
HIV-1 DNA is associated with HIV-1-specific T-cell immunity and HLA class I alleles. HIV-1 DNA levels presented according to whether the participant made a (**a**) CD4 or (**b**) CD8 interferon gamma ELISPOT response to HIV-1 Gag. Grey and white box and whisker plots represent Integrated and Total HIV-1 DNA, respectively, with diamonds indicating mean values: ‘Integrated No response' 3.72, ‘Integrated Response' 3.49, ‘Total No Response' 4.01, ‘Total Response' 3.77 log_10_ copies per million CD4 T cells, respectively. Significance determined using (**a**) *t*-tests and (**b**) linear regression. HIV-1 DNA reported as log_10_ copies per million CD4 T cells. Patient numbers: for ‘No Response' and ‘Response' for Total (*n*=33 and 60) and for Integrated (*n*=27 and 45), respectively. For 0, 1, 2, 3+ CD8+ T-cell responses for Total (*n*=22, 40, 25 and 20) and Integrated (*n*=22, 30, 23 and 17), respectively. (**c**) HLA Class I alleles ranked according to the median value of Total HIV-1 DNA, presented as box and whisker plots. Red and blue bars represent alleles associated with HIV-1 control and rapid progression, respectively. The horizontal black line represents the median value of the cohort and the box represents the inter-quartile range. The whiskers extend to the largest value within 1.5*IQR, with additional data points showing outliers. Patient numbers given in Supplementary Table 3. 0.01<*P<0.05, 0.001<**P<0.01, ***P<0.0001. Significance determined by Mann–Whitney test of target population against the rest of the patient samples.

To determine the relationship between the HIV-1 reservoir and HIV-1-specific T-cell immunity prior to therapy, we studied HIV-1 Gag-directed T-cell responses by ELISpot. Participants who made a CD4 T-cell ELISpot response had significantly lower mean HIV-1 DNA levels compared with those who did not (Fig. 1a; for total DNA *P*=0.018 (3.77 versus 4.01 log_10_ copies, respectively) and for Integrated *P*=0.046 (3.44 versus 3.72 log_10_ copies, respectively; *t*-test). Total (and to a lesser extent Integrated) HIV-1 DNA was inversely correlated with numbers of HIV-1 Gag-specific CD8 T-cell responses (*P*=0.046; *β*=−0.064; s.e. 0.032 and *P*=0.081, respectively; linear regression; Fig. 1b).

We then looked for associations with HLA Class I, the only consistent genetic association with HIV-1 progression^13^. For each HLA Class I allele in our cohort, the median value of Total HIV-1 DNA was calculated (numbers contributing to each HLA allele are in Supplementary Table 2). In Fig. 1c, HLA alleles were categorized as protective (red) or associated with rapid progression (blue) based on well-characterized associations with HIV-1 progression^14^. While individual favourable (for example, B*27 and B*57) and progressive (for example, B*35 and B*08) HLA Class I alleles were associated significantly with lower and higher Total HIV-1 DNA levels, respectively, we also found that, overall, participants with protective alleles (*n*=20) had significantly lower mean Total HIV-1 DNA levels than those with progression-associated alleles (*n*=55) (3.46 versus 4.05 log_10_ HIV-1 DNA copies per million CD4 T cells; *P*<0.001 (*t*-test)) (Fig. 1c).

### Co-variation of biomarkers at PHI

**Figure 2:**
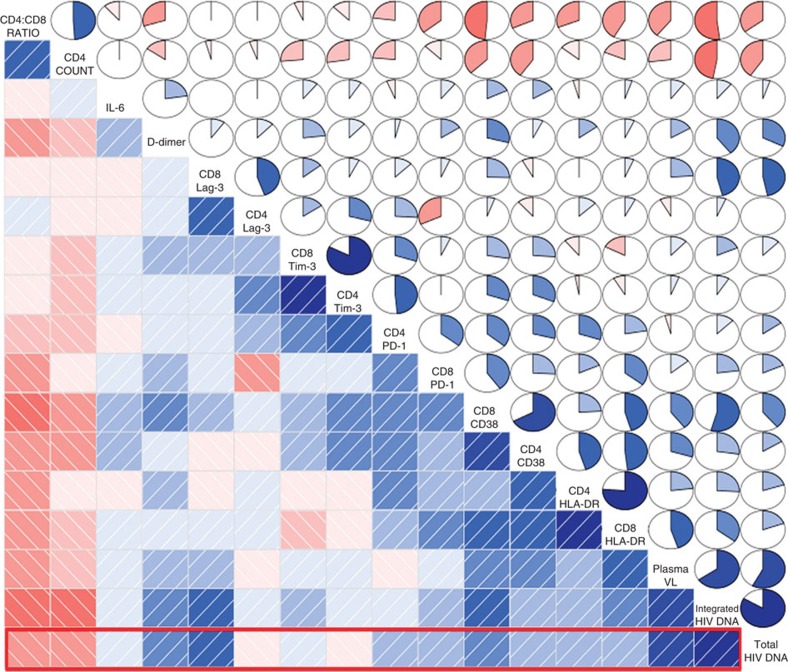
Correlogram of baseline virological and immunological variables. Heat maps and pie charts are used to indicate the strength of associations between potential biomarkers, with ordering determined by hierarchical clustering. Red indicates a negative correlation between the variables, blue a positive correlation. Size of pie and intensity of the colour indicates the strength of the association. Heat maps for associations with HIV-1 Total DNA are indicated with the red box.

Together, these data support a close interaction between T-cell immunity and the reservoir size in early HIV infection. An exploratory analysis was therefore carried out to illustrate co-variation within 17 immunological and reservoir-associated biomarkers, which were available at the baseline pre-therapy time-point. Results are presented graphically using a ‘correlogram'^15^ (Fig. 2) in which the order in which the variables are presented was determined by hierarchical clustering with optimal leaf ordering^16^. The heat map (lower triangle of the correlogram) indicates the direction (blue indicates a positive association and red indicates a negative association) and the strength of the correlation (intensity of colour). The same information is also conveyed in the series of pie charts (upper triangle), where the strength of the correlation is indicated by both the size of the pie slice and intensity of colour.

In Fig. 2, the correlogram shows negative associations between CD4 T-cell count and all markers of viral nucleic acid and immune function (except IL-6). PD-1, CD38 and HLA-DR expression on CD4 and CD8 T cells, as represented by the deeper blue shaded region, cluster together with similar positive correlations. The correlogram shows that eight immunological variables: D-dimer, Lag-3 (CD8 only), HLA-DR (CD4, CD8), PD-1 (CD4, CD8), CD38 (CD4, CD8), showed the strongest correlations with Total HIV-1 DNA and were selected for further analysis.

### Correlates of Total HIV-1 DNA pre-ART and at TI

As Total HIV-1 DNA was a predictor of time to plasma viral load (VL) rebound in SPARTAC^11^, we wished to explore the joint association of these biomarkers with HIV-1 DNA in a regression analysis. In addition to the eight variables identified in the correlation analysis, we included three clinical markers associated with HIV-1 DNA: plasma VL, CD4 T-cell count and CD4/CD8 T-cell ratio, which were also evident in the correlogram. Plasma VL, CD4 T-cell count and CD4/CD8 ratio were all associated significantly (*P*<0.001; linear regression) with HIV-1 DNA levels, although in multivariable models (adjusting for just these measures) the CD4/CD8 ratio was no longer significant (Table 2; Model A). We found strong, significant correlations with biomarkers for CD4 and CD8 T-cell activation and exhaustion, as well as D-dimer (Table 2, Model B). In a multivariable model (*n*=78), CD8 CD38, CD8 Lag-3 and D-dimer retained a significant association with HIV-1 Total DNA. (These results are not overly impacted by adjusting for multiple comparisons (Supplementary Table 3); significance (at *P*<0.05) was lost for HLA-DR). In a further model including plasma VL and CD4 T-cell count, the only biomarker to retain a significant association with HIV-1 DNA was CD8 Lag-3 (Table 2, Model C).

Many of the immunological associations present pre-ART were not preserved following ART. After 48 weeks of ART (at the point of TI) the only statistical associations with contemporaneously measured Total HIV-1 DNA were unspliced cell-associated HIV-1 RNA and percentage HLA-DR on CD4 T cells (Supplementary Table 4).

### Predictors of time to plasma HIV-1 viral load rebound

Would biomarkers associated with HIV-1 DNA also predict time to VL rebound after TI? Although sample limitation restricted the analysis of all biomarkers in all 47 patients, we could find no strong evidence for any biomarker (other than Total HIV-1 DNA) measured at TI predicting viral rebound. CD4 T-cell ELISpots measured at baseline or TI did not predict time to rebound (Supplementary Fig. 1).

**Figure 3:**
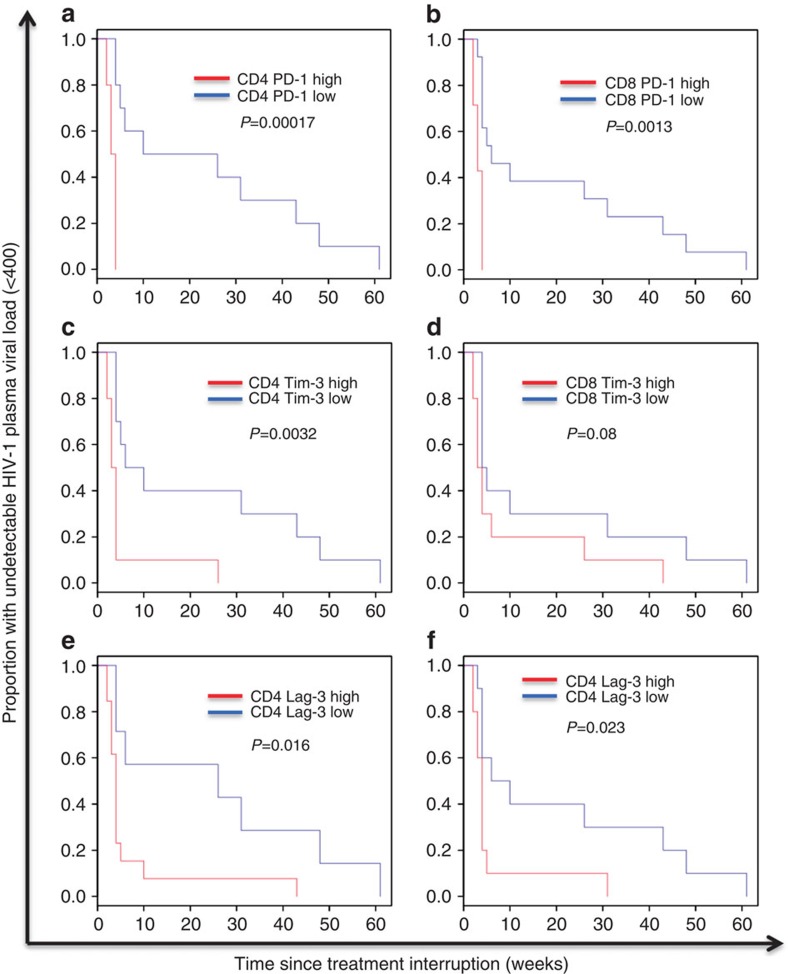
Expression of T-cell exhaustion markers measured at baseline and survival analyses for time to viral load rebound. For each Kaplan–Meier analysis, the variable was stratified into ‘high' and ‘low' at the median level. Median values were 4.87% and 8.15% for PD-1, 14.65% and 11.85% for Tim-3, and 7.60% and 14.15% for Lag-3 on CD8 and CD4 T cells, respectively. *P* values determined by log rank test. *N*=20 for each analysis. (**a**,**b**) CD4 and CD8 PD-1 expression, respectively. (**c**,**d**) CD4 and CD8 Tim-3, respectively. (**e**,**f**) CD4 and CD8 Lag-3, respectively.

However, pre-therapy levels of T-cell exhaustion were strongly associated with time to VL rebound >400 copies per ml after TI. In survival analyses dividing the cohort at median values, expression on CD4 and CD8 T cells of PD-1 (*P*=0.00017, *P*=0.0013), Tim-3 (*P*=0.0032, *P*=0.08) and Lag-3 (*P*=0.016, *P*=0.023), respectively, predicted viral rebound to 400 copies per ml (log rank test) (Fig. 3a–f). These findings were supported by Cox models adjusting for HIV-1 DNA levels (Table 3; Supplementary Table 5). Adjusting for multiple comparisons (Supplementary Table 6) had no impact on the significance for PD-1, although Lag-3 expression was no longer significant. When using a VL cut-off for rebound of 50 RNA copies per ml, Tim-3 (*P*<0.001 for CD4; *P*=0.0067 for CD8 T cells) and PD-1 (*P*=0.023 for CD4 T cells) were predictive of time to rebound (Supplementary Table 7).

**Figure 4:**
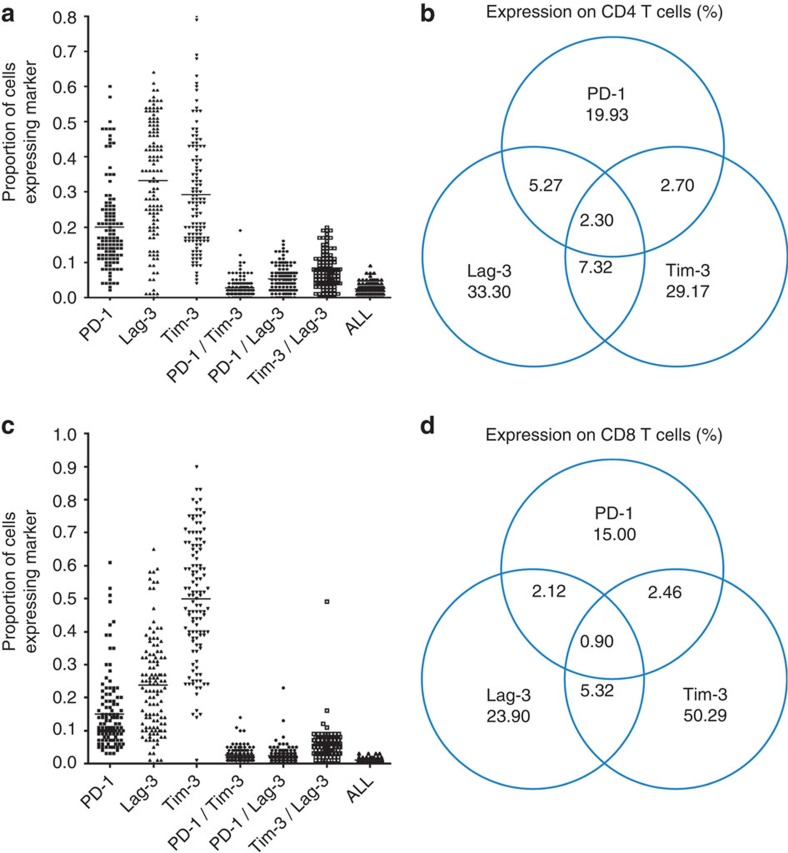
Co-expression of immune checkpoint markers. Data plots and Venn diagrams to show expression of one, two or three exhaustion markers (Tim-3, Lag-3 and PD-1) at pre-therapy baseline on CD4 (*n*=120) (**a**,**b**) and CD8 (*n*=118) (**c**,**d**) T cells. Figures represent the percentage contribution to different combinations of expression of the three markers with overall expression normalized to 100%.

No other baseline measures (including markers of activation, IL-6 and D-dimer) were significantly associated with time to rebound. While PD-1 and Lag-3 correlated with HIV-1 DNA levels, Tim-3 did not, suggesting a possible separate mechanism of action. When adjusting for other exhaustion markers, Tim-3 expression on CD4 T cells and CD8 T cells was the only marker retaining significance for all comparisons (Supplementary Table 5). PD-1 and Lag-3 retained significance for expression on CD8 T cells only. Of note, none of PD-1, Tim-3 or Lag-3 predicted time to VL rebound when measured at TI (Supplementary Table 8). Although all badged as ‘exhaustion markers' (or ‘immune checkpoint markers), it is interesting to note that Tim-3, PD-1 and Lag-3 were predominantly expressed on separate cells. While all three markers were widely expressed on their own on CD4 (range 19.93–33.30%) and CD8 (range 15.00–50.29%) T cells, co-expression of any two markers was limited (Fig. 4).

## Discussion

ART in PHI can, in some cases, induce a state of virological remission or PTC. In the search for a cure for HIV-1 infection, it is important to understand the underlying mechanisms that produce PTC and assess the possibility of identifying patients most likely to achieve PTC using an algorithm of biomarkers.

The SPARTAC RCT was not designed to identify biomarkers that predict remission, yet the incorporation of a randomly allocated TI after 48 weeks of ART provided a unique opportunity due to the numerous virological and immunological studies undertaken within the protocol. TI studies have been set back by the increased mortality witnessed in the SMART study^17^, yet newer study designs in which ART is recommenced based on viral detection in plasma rather than CD4 decline may provide a safer option. Accordingly, a number of studies are currently being planned to assess the impact of ART on virological remission through intensively monitored TIs, with the assessment of biomarkers being a key endpoint. This analysis of SPARTAC therefore provides preliminary critical data to help inform future larger clinical studies and cohorts.

Although no long-term PTC was seen in this group of patients, we did find a prolonged period of aviraemia post TI—1 year after stopping ART 14% of participants were still undetectable^5^. In SPARTAC, Total (but not Integrated) HIV-1 DNA measurements at baseline and week 48 helped stratify patients according to their viral-rebound kinetics^11^. We now show that—prior to ART—numerous measures of T-cell immunity (both HIV-1 specific and non-specific) measured in over 150 individuals with PHI correlated closely with Total HIV-1 DNA levels, and we speculated that these might also be predictors of time to viral rebound. Of note, T-cell immunity and HIV-1 DNA are predictors of clinical progression, as determined by CD4 T-cell count decline and viraemia^11^,^18^,^19^,^20^. However, these associations did not persist in relation to predicting VL rebound, suggesting that these two processes—time to rebound and time to clinical progression—have different mechanisms. Indeed, in SPARTAC, these two time intervals after TI did not correlate (Supplementary Fig. 3). While there is plenty of evidence to suggest that the progression is immune mediated, it is increasingly proposed—and supported by mathematical modelling^21^,^22^—that rebound is stochastic, or random^23^, that is, the initiation of viral transcription in a latently infected cell at levels adequate to result in plasma viraemia may not be initially governed by the immune response.

These data suggest that the size of the reservoir is determined by T-cell-mediated immunity in early HIV-1 infection, reflected in the number of associations between HIV-1 DNA and immune biomarkers at baseline. Once ART is commenced and viral replication is suppressed, the CD4 T-cell count recovers and the HIV-1 reservoir (in reality, an unknown entity for which we have limited surrogate measures) declines. At the point of TI, two events can occur. (i) viraemia returns; and (ii) absolute CD4 T-cell numbers fall until a point at which ART needs to be reintroduced. We hypothesize that these two processes are determined by different mechanisms: (i) stochastic and dependent on the size of the reservoir size; and (ii) mediated by HIV-1-specific immune responses after viraemia has returned. Accordingly, it is possible that studies describing T-cell-mediated mechanisms driving ‘elite controllers' may have little to offer our understanding of how to induce PTC or HIV-1 cure. This model may be supported by our finding that T-cell exhaustion markers at baseline (48 weeks prior to TI) predict time to viral rebound, whereas the same markers measured at the time of TI do not. (A caveat to this finding is that different antibody clones were used for the surface fluorescence-activated cell sorting (FACS) staining at baseline and at week 48 and, due to the possibility of different affinities, this is a possible confounder which needs to be explored in future studies.)

The highly significant impact of the markers PD-1, Tim-3 and Lag-3 is intriguing, especially in view of the lack of association for markers of activation (HLA-DR, CD38), which might be expected to associate with rebound. PD-1 has been widely reported to be upregulated on exhausted T cells in chronic viral infections such as cytomegalovirus, simian immunodeficiency virus (SIV) and HIV, in a process that is reversible through blockade of PD-1 with its ligand. Accordingly, some investigators have suggested that PD-1 is a marker of early exhaustion or even activation—the latter being supported by its clustering with HLA-DR and CD38 in the correlogram. Despite the identification of PD-1, Tim-3 and Lag-3 as potential biomarkers for viral rebound, this does not in itself prove that they are mechanistic. There is much interest in the therapeutic potential of blocking PD-1 (either directly or by antagonizing the PD-1 ligand), and even in the context of targeting the HIV-1 reservoir. Of note, the combination of anti-PD-1 (Nivolumab) and anti-CTLA-4 (Ipilimumab) monoclonal antibodies have shown encouraging results in clinical trials in untreated melanoma^24^, and one might speculate that this enhancement in T-cell function may also be applicable for targeting the HIV-1 reservoir.

Tim-3 has been associated with T-cell exhaustion (loss of functionality and decreased proliferation) in the context of HIV-1 infection^25^. Tim-3 expression on both CD4 and CD8 T cells is elevated in HIV+ve individuals compared with controls, correlates with VL and is associated with disease progression. As for PD-1, blocking the interaction of Tim-3 with its ligand can restore functionality. Interestingly, in the same study it was shown that PD-1 and Tim-3 identify distinct populations of cells, rather than being co-expressed on the same dysfunctional T cells, which we also found in our patients. This might help explain why they both predict time to rebound and yet both are not associated with HIV-1 DNA levels. Increased understanding of which cells express these markers and how they contribute to the HIV-1 reservoir could be important in developing new HIV-1 cure strategies. This will need to be addressed in larger studies carefully designed for this purpose.

The role of Lag-3 (CD223) in HIV-1 infection is less clear, although its upregulation has been associated with disease progression and functional T-cell exhaustion^26^. Lag-3 is structurally similar to the CD4 receptor and is a ligand for MHC Class II, and part of the Ig superfamily on memory T cells^27^. Mouse models have suggested some synergy between PD-1 and Lag-3 (ref. 28), although it is interesting that we only find limited co-expression in our data. However, now that the proof-of-efficacy has been demonstrated in cancer trials for combination immune checkpoint blockade^24^, it will be important to conduct further studies to explore how this applies to HIV latency.

In summary, this analysis of SPARTAC participants undergoing TI after 48 weeks of ART reveals potential new biomarkers that should be considered in larger studies exploring PTC. The correlations between measures of T-cell-mediated immunity and HIV-1 DNA prior to therapy make a case for the reservoir size being determined by T-cell function early in infection. The relationship between immune responses and the duration of PTC at TI is much less clear, but the association with high levels of T-cell exhaustion might help shed light on why some latently infected cells reactivate and others do not. The work should help in future studies to stratify patients for PTC, with the ultimate aim to determine the mechanism of PTC, and identify new targets for HIV-1 cure research.

## Methods

### Participants and trial design

SPARTAC^10^ was an international RCT enroling adults with PHI within 6 months of a last negative, equivocal or incident HIV-1 test. All participants gave written informed consent. Time of seroconversion was estimated as the midpoint of last negative/equivocal and first positive tests, or date of incident test. Participants were randomized to receive ART for 48 weeks (ART-48), 12 weeks (ART-12) or no therapy (standard of care).

Participants for this sub-study were those infected with subtype B HIV-1 and for whom adequate samples were available. For those in the analysis of viral rebound at TI, we only considered participants in the ART-48 arm who had viral load suppression (<50 HIV-1 copies per ml; Chiron bDNA, Bayer) at the point of stopping ART (Table 1). Peripheral blood mononuclear cells (PBMC) were available for analysis in all participants at baseline, regardless of treatment allocation. Participants randomized to the ART-48 arm were sampled at week 48 immediately prior to TI. Viral remission was defined as maintaining a HIV-1 RNA viral load <400 copies per ml after stopping ART.

### Ethics statement

The SPARTAC trial, and the immunological and virological analyses performed in this study were approved by the following authorities: the Medicines and Healthcare products Regulatory Agency (UK), the Ministry of Health (Brazil), the Irish Medicines Board (Ireland), the Medicines Control Council (South Africa) and the Uganda National Council for Science and Technology (Uganda). It was also approved by the following ethics committees in the participating countries: the Central London Research Ethics Committee (UK), Hospital Universitário Clementino Fraga Filho Ethics in Research Committee (Brazil), the Clinical Research and Ethics Committee of Hospital Clinic in the province of Barcelona (Spain), the Adelaide and Meath Hospital Research Ethics Committee (Ireland), the University of Witwatersrand Human Research Ethics Committee, the University of Kwazulu-Natal Research Ethics Committee and the University of Cape Town Research Ethics Committee (South Africa), Uganda Virus Research Institute Science and ethics committee (Uganda), the Prince Charles Hospital Human Research Ethics Committee and St Vincent's Hospital Human Research Ethics Committee (Australia) and the National Institute for Infectious Diseases Lazzaro Spallanzani, Institute Hospital and the Medical Research Ethics Committee, and the ethical committee of the Central Foundation of San Raffaele, MonteTabor (Italy). All participants signed a written informed consent.

### Measurement of HIV-1 DNA

CD4 T cells were enriched from frozen PBMC samples by negative selection (Dynabeads) to a purity of >97%. CD4 T-cell DNA was extracted (Qiagen) and used as input DNA for PCR. Cell copy number and Total HIV-1 DNA levels were quantified in triplicate using previously published assays^29^,^30^. Integrated HIV-1 was measured using an assay based on that previously published^31^ but with some minor modifications as described elsewhere^11^.

### Unspliced cell-associated HIV-1 RNA Transcript quantitation

RNA was isolated using the Qiagen AllPrep DNA/RNA Mini kit as per the manufacturer's recommendations with the addition of an on-column double DNase digestion. The RNA assay was a modified version of those previously described^32^,^33^,^34^. Briefly, complementary DNA was subjected to two rounds of PCR using semi-nested primers. The first reaction was a 15-cycle end-point reaction using MH535 (5′-AACTAGGGAACCCACTGCTTAAG-3′) and SL20 (5′-TCTCCTTCTAGCCTCCGCTAGTC-3′) primers at a concentration of 400 nM each^35^ as previously described^32^,^33^,^34^. The second reaction was a 40-cycle quantitative reaction (forward primer 5′-TAAAGCTTGCCTTGAGTGCT, SL20 and the probe FAM—5′-AGTRGTGTGTGCCCGTCTGTTG-3′—BHQ-1), performed using a Roche Lightcycler 480. The HIV standard was generated by *in vitro* transcribing the plasmid Sp5-NL4.3 (generously provided by the lab of D. Purcell) using the RiboMax Large Scale SP6 RNA production System (Promega). HIV RNA standards were diluted in 10 ng μl^−1^ uninfected PBMC RNA. HIV measurements were normalized to input cellular RNA using the 18S gene with the Amplifluor Human/Mouse 18S rRNA Primer Set (FAM labelled; Millipore, Cat No. SCR593). All samples were negative when run without reverse transcriptase as a control.

### CD8 and CD4 T-cell ELISpot assays

Quantification of the HIV-1-specific CD8 response was performed by IFN-γ ELISpot analysis^20^. Responses were determined to subtype B consensus HIV-1 Gag overlapping peptides covering the entire Gag protein (123 15mers overlapping by 11 amino acids; AIDS Research and Reference Reagent Program, NIH). CD4 T-cell IFN-γ ELISpot assays were carried out in real time in UK subtype B participants on fresh blood samples. Methods are described elsewhere, but essentially, CD4 T-cell frequencies against HIV-1 subtype B p24 were determined using IFN-γ ELISpot using fresh CD8 T-cell-depleted PBMCs. CD4 samples were analysed in duplicate and results were expressed as spot forming units per 10^6^ CD8-depleted peripheral blood leucocytes. ELISpot data analysis is described elsewhere^20^.

### Multiparameter FACS

Thawed PBMCs from healthy individuals (controls) and HIV-1-infected patients (SPARTAC) were stained with two panels of antibodies. For markers of activation, we used CD25 (PE, Miltenyi clone 3G10, 2 μl per 50 μl), CD38 (PE-Vio770; Miltenyi clone IB6, 1 μl per 50 μl), CD69 (FITC, Miltenyi clone FN50, 1 μl per 50 μl) and HLA-DR (PerCP, Miltenyi clone AC122, 1 μl per 50 μl). For markers of exhaustion, we used Tim-3 (FITC, eBioscience clone F38-2E2; 0.05 μg per 100 μl), Lag-3 (PerCP-eF710, eBioscience clone 3D5223H, 0.024 μg per 100 μl), PD-1 (PE-Cy7, eBioscience clone eBioJ105, 0.2 μg per 100 μl) and TIGIT (PE, eBioscience clone MBSA43, 0.05 μg per 100 μl). Both panels also included the anchor markers CD3 (VioBlue, Miltenyi clone BW264/56, 0.5 μl per 50 μl), CD4 (VioGreen, Miltenyi clone VIT4, 2 μl per 50 μl), CD8 (APC, Miltenyi cloneBW135/80, 1 μl per 50 μl) and a Live/Dead marker, Near IR-APC-Cy7 (1:300 dilution). Cells were run on a MAQSquant and analysed with FlowJO software. The gating strategy is present in Supplementary Fig. 2. Results are reported throughout as percentages of cells expressing the marker in question. Different antibody clones were used for the surface FACS staining for Tim-3 (PE, R&D clone 344823, 0.05 μg per 100 μl), PD-1 (APC, eBiosciences clone MIH4, 0.5 μg per 100 μl) and Lag-3 (FITC, LifeSpan Biosciences clone 17B4, 2 μg per 100 μl) at week 48 and, due to the possibility of different affinities, this is a possible confounder which needs to be explored in future studies.

### ELISAs

Interleukin-6 (IL-6) and D-dimer were quantified using commercial assays (Quantikine HS600B IL-6 immunoassay (R&D Systems, Minneapolis, Minnesota, USA) and Innovance D-dimer (Siemens Healthcare Diagnostics, Tarrytown, New York, USA)), as described elsewhere^36^.

### Statistical analysis

Tests between grouped variables and transformed log_10_ DNA levels were performed using *t*-tests and linear regression. Differences between HIV-1 DNA levels for individual HLA Class I alleles and other HLA alleles were examined using Mann–Whitney tests. Comparisons of HIV-1 DNA levels between patients carrying ‘protective' and ‘progressive' HLA alleles used *t*-tests. A correlogram^15^ was used to display the Pearson correlations between the biomarkers. Variables with similar correlations are grouped together. The direction and size of the correlations are illustrated by heat colour and pie charts. The order of the biomarkers within the correlogram is determined by hierarchical clustering: there are 2^*n*-1^ (2^17-1^) possible orderings of the heat map, we use the optimal leaf order algorithm^16^ to minimize the difference between adjacent biomarkers. The R package correlogram was used to produce the image. Linear regression was carried out between baseline HIV-1 Total DNA and variables selected from the correlogram. The optimized multivariable model in Table 1B is constructed stepwise in both directions to minimize AIC using the R step function. Time to viral rebound was examined with Kaplan–Meier curves, and Cox proportional hazards models. The proportional hazard assumption was tested by examining Schoenfeld residuals using the cox.zph function from the R survival package. Time to VL rebound was defined as the time from TI (at week 48) to the first of more than one consecutive detectable VL>400 copies per ml. For T-cell exhaustion markers, time to rebound was also analysed for >50 copies per ml. All statistics were calculated using R version 3.1.0. Plots were drawn with R and Prism.

## Additional information

**How to cite this article:** Hurst, J. *et al*. Immunological biomarkers predict HIV-1 viral rebound after treatment interruption. *Nat. Commun.* 6:8495 doi: 10.1038/ncomms9495 (2015).

## Supplementary Material

Supplementary Information Supplementary Figures 1-3, Supplementary Tables 1-8 and Supplementary Note 1

## Figures and Tables

**Table 1:** Biomarkers and methods of measurement.

**Biomarker ‘class'**	**Biomarker**	**Sample**	**Assay platform**	**Time-point analysed week (*****n***)
Clinical	CD4 cell count	Blood	Flow cytometry	0 (154)48 (47)
	Plasma viral load	Plasma	Commercial assay	0 (154)48 (47)
	CD4/CD8 ratio	Blood	Flow cytometry	0 (154)48 (47)
Viral DNA	HIV-1 DNA (Total)	CD4 T cells	qPCR	0 (154)48 (47)
	HIV-1 DNA (Integrated)	CD4 T cells	qPCR	0 (111)48 (47)
	Cell-associated unspliced HIV-1 RNA	CD4 T cells	qPCR	0 (0)48 (27)
HIV-1-specific T-cell immunity	CD8 ELISpot	CD8 T cells	ELISpot	0 (107)48 (0)
	CD4 ELISpot	CD4 T cells	ELISpot	0 (93)48 (32)
T-cell activation	HLA-DR	CD4/CD8 T cells	Flow cytometry	0 (89)48 (40)
	CD38	CD4/CD8 T cells	Flow cytometry	0 (89)48 (40)
	CD25	CD4/CD8 T cells	Flow cytometry	0 (0)48 (40)
	CD69	CD4/CD8 T cells	Flow cytometry	0 (0)48 (40)
T-cell exhaustion	PD-1	CD4/CD8 T cells	Flow cytometry	0 (78)48 (37)
	Lag-3	CD4/CD8 T cells	Flow cytometry	0 (78)48 (37)
	Tim-3	CD4/CD8 T cells	Flow cytometry	0 (78)48 (37)
	TIGIT	CD4/CD8 T cells	Flow cytometry	0 (0)48 (37)
Soluble markers of immune activation	IL-6	Plasma	ELISA	0 (143)48 (42)
	D-dimer	Plasma	ELISA	0 (137)48 (40)

ELISA, enzyme-linked immunosorbent assay; qPCR, quantitative PCR.

Details of 18 biomarkers studied including tissue source, assay platform and time-point of analysis and total number of participant samples available at each time-point. Not all samples were available at all time-points.

**Table 2:** Biomarkers associated with total HIV-1 DNA at PHI.

**A**	**Baseline variable**	**Simple linear model (*****β***; s.e.; *P*-value)*	**Multivariable regression model A (*****β***; s.e.; *P*-value)*
	CD4/CD8 T-cell ratio	−0.904; 0.15; <0.001	−0.002; 0.16; 0.99
	CD4 T-cell count	−14.9; 0.425; <0.001	−8.56; 2.29; <0.001
	log_10_ viral load	4.24; 0.425; <0.001	3.69; 0.52; <0.001
**B**		**Simple linear model (*****β***; s.e.; *P*-value)*	**Multivariable regression model B (*****β***; s.e.; *P*-value)*
	CD8+ CD38+†	0.017; 0.003;<0.001	0.012; 0.003;<0.001
	CD4+ CD38+†	0.007; 0.004; 0.055	—‡
	CD8+PD-1†	0.057; 0.017; 0.002	—‡
	CD4+PD-1†	0.020; 0.014; 0.169	—‡
	CD8+HLA-DR†	0.010; 0.003; 0.005	—‡
	CD4+HLA-DR†	0.017; 0.008; 0.041	—‡
	CD8+Lag-3†	0.012; 0.004; 0.002	0.019; 0.009; 0.04
	D-dimer	0.034; 0.010; <0.001	0.536; 0.236; 0.026
**C**			**Multivariable regression model C (*****β***; s.e.; *P*-value)*
	CD4 T-cell count		−8.23; 2.04; <0.001
	log_10_ viral load		3.047; 0.45;<0.001
	CD8 CD38†		0.002; 0.002; 0.329
	CD8 Lag-3†		0.017; 0.007; 0.014
	D-dimer		0.28; 0.17; 0.1

PHI, primary HIV-1 infection.

Linear regression models showing associations at baseline with log_10_ Total HIV-DNA. Model A: *N*=76, multivariable linear regression model with log_10_ Total HIV-1 DNA as the dependent variable adjusting for the 3 clinical measures. Model B: *N*=78, forward and backwards stepwise-optimized linear regression model with log_10_ Total HIV-1 DNA as the dependent variable, considering eight immunological variables initially selected on the basis of having a positive correlation with Total DNA in the correlogram. Model C: *N*=76, multivariable linear regression at baseline with log_10_ (Total HIV-1) as the dependent variable including clinical and immunological variables.

^*^β, regression coefficients; s.e. and *P* values.

^†^Expression on T cells.

^‡^Not selected for the final optimized model.

**Table 3:** Baseline biomarkers predicting viral rebound after TI.

**Biomarker expression on T cells*****N*****=20**	**Unadjusted HR (CI)** ***P***-**value**	**Adjusted for baseline HIV-1 DNAHR (CI)** ***P***-**value**	**Adjusted for week 48 HIV-1 DNAHR (CI)** ***P***-**value**
PD-1 CD4+	1.35 (1.07–1.71)*P*=0.011	1.46 (1.06–1.85)*P*=0.016	1.42 (1.10–1.84)*P*=0.0074
PD-1 CD8+	1.15 (1.02–1.32)*P*=0.029	1.37 (0.96–1.35)*P*=0.15	1.15 (1.01–1.32)*P*=0.034
Tim-3 CD4+	1.25 (1.12–1.40)*P*<0.001	1.36 (1.16–1.60)*P*=0.009	1.27 (1.12–1.14)*P*<0.001
Tim-3 CD8+	1.11 (1.04–1.20)*P*=0.0036	1.15 (1.06–1.26)*P*=0.0011	1.11 (1.03–1.20)*P*=0.0034
Lag-3 CD4+	1.08 (1.03–1.15)*P*=0.0036	1.082 (1.02–1.15)*P*=0.0066	1.09 (1.028–1.14)*P*=0.0035
Lag-3 CD8+	1.104 (1.03–1.19)*P*=0.0093	1.129 (0.99–1.28)*P*=0.056	1.24 (1.02–1.24)*P*=0.015

CI, confidence interval; HR, hazard ratio; TI, treatment interruption; VL, viral load.

Cox models reporting biomarkers measured at pre-therapy baseline (week 0) and time to plasma VL rebound (>400 copies per ml) after TI, 48 weeks later. Columns represent three models: unadjusted, adjusting for baseline HIV-1 Total DNA and adjusting for week 48 HIV-1 Total DNA.

## References

[b1] Palella F. J. J. . Declining morbidity and mortality among patients with advanced human immunodeficiency virus infection. HIV outpatient study investigators. N. Engl. J. Med. 338, 853–860 (1998). 9516219 10.1056/NEJM199803263381301

[b2] Wada N. . Cause-specific mortality among HIV-infected individuals, by CD4(+) cell count at HAART initiation, compared with HIV-uninfected individuals. AIDS 28, 257–265 (2014). 24105030 10.1097/QAD.0000000000000078 PMC4164055

[b3] Samji H. . Closing the gap: increases in life expectancy among treated HIV-positive individuals in the United States and Canada. PLoS ONE 8, e81355 (2013). 24367482 10.1371/journal.pone.0081355 PMC3867319

[b4] Finzi D. . Identification of a reservoir for HIV-1 in patients on highly active antiretroviral therapy. Science 278, 1295–1300 (1997). 9360927 10.1126/science.278.5341.1295

[b5] Stöhr W. . Duration of HIV-1 viral suppression on cessation of antiretroviral therapy in primary infection correlates with time on therapy. PLoS ONE 8, e78287 (2013). 24205183 10.1371/journal.pone.0078287 PMC3808338

[b6] Saez-Cirion A. . Post-treatment HIV-1 controllers with a long-term virological remission after the interruption of early initiated antiretroviral therapy ANRS VISCONTI Study. PLoS Pathog. 9, e1003211 (2013). 23516360 10.1371/journal.ppat.1003211 PMC3597518

[b7] Lodi S. . Immunovirologic control 24 months after interruption of antiretroviral therapy initiated close to HIV seroconversion. Arch. Intern. Med. 172, 1252–1255 (2012). 22826124 10.1001/archinternmed.2012.2719

[b8] Goujard C. . HIV-1 control after transient antiretroviral treatment initiated in primary infection: role of patient characteristics and effect of therapy. Antivir. Ther. (Lond.) 17, 1001–1009 (2012). 22865544 10.3851/IMP2273

[b9] Goulder P. J. & Walker B. D. HIV and HLA class I: an evolving relationship. Immunity 37, 426–440 (2012). 22999948 10.1016/j.immuni.2012.09.005 PMC3966573

[b10] SPARTAC Trial Investigators. . Short-course antiretroviral therapy in primary HIV infection. N. Engl. J. Med. 368, 207–217 (2013). 23323897 10.1056/NEJMoa1110039 PMC4131004

[b11] Williams J. P. . HIV-1 DNA predicts disease progression and post-treatment virological control. Elife 3, e03821 (2014). 25217531 10.7554/eLife.03821 PMC4199415

[b12] Fidler S. . Short-Course Antiretroviral Therapy in Primary HIV Infection. N. Engl. J. Med. 368, 207–217 (2013). 23323897 10.1056/NEJMoa1110039 PMC4131004

[b13] Fellay J. . A whole-genome association study of major determinants for host control of HIV-1. Science 317, 944–947 (2007). 17641165 10.1126/science.1143767 PMC1991296

[b14] Pereyra F. . The major genetic determinants of HIV-1 control affect HLA class I peptide presentation. Science 330, 1551–1557 (2010). 21051598 10.1126/science.1195271 PMC3235490

[b15] Friendly M. Corrgrams: exploratory displays for correlation matrices. Am. Stat. 56, 316–324.

[b16] Bar-Joseph Z., Gifford D. K. & Jaakkola T. S. Fast optimal leaf ordering for hierarchical clustering. Bioinformatics 17, (suppl 1): S22–S29 (2001). 11472989 10.1093/bioinformatics/17.suppl_1.s22

[b17] El-Sadr W. M. . CD4+ count-guided interruption of antiretroviral treatment. N. Engl. J. Med. 355, 2283–2296 (2006). 17135583 10.1056/NEJMoa062360

[b18] Kiepiela P. . CD8+ T-cell responses to different HIV proteins have discordant associations with viral load. Nat. Med. 13, 46–53 (2007). 17173051 10.1038/nm1520

[b19] Tierney C. . Prognostic value of baseline human immunodeficiency virus type 1 DNA measurement for disease progression in patients receiving nucleoside therapy. J. Infect. Dis. 187, 144–148 (2003). 12508159 10.1086/345870

[b20] Frater J. . HIV-1 specific CD4 responses in primary HIV-1 infection predict disease progression in the SPARTAC trial. AIDS 28, 699–708 (2014). 24549145 10.1097/QAD.0000000000000130

[b21] Conway J. M. & Coombs D. A stochastic model of latently infected cell reactivation and viral blip generation in treated HIV patients. PLoS Comput. Biol. 7, e1002033 (2011). 21552334 10.1371/journal.pcbi.1002033 PMC3084212

[b22] Wang S. & Rong L. Stochastic population switch may explain the latent reservoir stability and intermittent viral blips in HIV patients on suppressive therapy. J. Theor. Biol. 360, 137–148 (2014). 25016044 10.1016/j.jtbi.2014.06.042

[b23] Rouzine I. M., Razooky B. S. & Weinberger L. S. Stochastic variability in HIV affects viral eradication. Proc. Natl Acad. Sci. USA 111, 13251–13252 (2014). 25201951 10.1073/pnas.1413362111 PMC4169906

[b24] Larkin J. . Combined nivolumab and ipilimumab or monotherapy in untreated melanoma. N. Engl. J. Med. 373, 23–24 (2015). 26027431 10.1056/NEJMoa1504030 PMC5698905

[b25] Jones R. B. . Tim-3 expression defines a novel population of dysfunctional T cells with highly elevated frequencies in progressive HIV-1 infection. J. Exp. Med. 205, 2763–2779 (2008). 19001139 10.1084/jem.20081398 PMC2585847

[b26] Tian X. . The upregulation of LAG-3 on T cells defines a subpopulation with functional exhaustion and correlates with disease progression in HIV-infected subjects. J. Immunol. 194, 3873–3882 (2015). 25780040 10.4049/jimmunol.1402176

[b27] Larsson M. . Molecular signatures of T-cell inhibition in HIV-1 infection. Retrovirology 10, 31 (2013). 23514593 10.1186/1742-4690-10-31 PMC3610157

[b28] Okazaki T. . PD-1 and LAG-3 inhibitory co-receptors act synergistically to prevent autoimmunity in mice. J. Exp. Med. 208, 395–407 (2011). 21300912 10.1084/jem.20100466 PMC3039848

[b29] Duncan C. J. . High-multiplicity HIV-1 infection and neutralizing antibody evasion mediated by the macrophage-T Cell virological synapse. J. Virol. 88, 2025–2034 (2013). 24307588 10.1128/JVI.03245-13 PMC3911534

[b30] Williams J. . Low copy target detection by Droplet Digital PCR through application of a novel open access bioinformatic pipeline, ‘definetherain'. J. Virol. Methods 202, 46–53 (2014). 24598230 10.1016/j.jviromet.2014.02.020 PMC4003534

[b31] Liszewski M. K., Yu J. J. & O'Doherty U. Detecting HIV-1 integration by repetitive-sampling Alu-gag PCR. Methods 47, 254–260 (2009). 19195495 10.1016/j.ymeth.2009.01.002 PMC2862469

[b32] Pasternak A. O. . Highly sensitive methods based on seminested real-time reverse transcription-PCR for quantitation of human immunodeficiency virus type 1 unspliced and multiply spliced RNA and proviral DNA. J. Clin. Microbiol. 46, 2206–2211 (2008). 18463204 10.1128/JCM.00055-08 PMC2446885

[b33] Pasternak A. O. . Cellular levels of HIV unspliced RNA from patients on combination antiretroviral therapy with undetectable plasma viremia predict the therapy outcome. PLoS ONE 4, e8490 (2009). 20046870 10.1371/journal.pone.0008490 PMC2795168

[b34] Lewin S. R. . Use of real-time PCR and molecular beacons to detect virus replication in human immunodeficiency virus type 1-infected individuals on prolonged effective antiretroviral therapy. J. Virol. 73, 6099–6103 (1999). 10364365 10.1128/jvi.73.7.6099-6103.1999 PMC112674

[b35] Saleh S. . Expression and reactivation of HIV in a chemokine induced model of HIV latency in primary resting CD4+ T cells. Retrovirology 8, 80 (2011). 21992606 10.1186/1742-4690-8-80 PMC3215964

[b36] Hamlyn E. . Interleukin-6 and D-dimer levels at seroconversion as predictors of HIV-1 disease progression. AIDS 28, 869–874 (2014). 24300544 10.1097/QAD.0000000000000155

