# Supplementary material for: Immunological biomarkers predict HIV-1 viral rebound after treatment interruption
Source: Nat Commun. 2015 Oct 9;6:8495. doi: 10.1038/ncomms9495 (PMC4633715; doi:10.1038/ncomms9495)
Supplement: Supplementary Information — Supplementary Figures 1-3, Supplementary Tables 1-8 and Supplementary Note 1 [file ncomms9495-s1.pdf]

a.

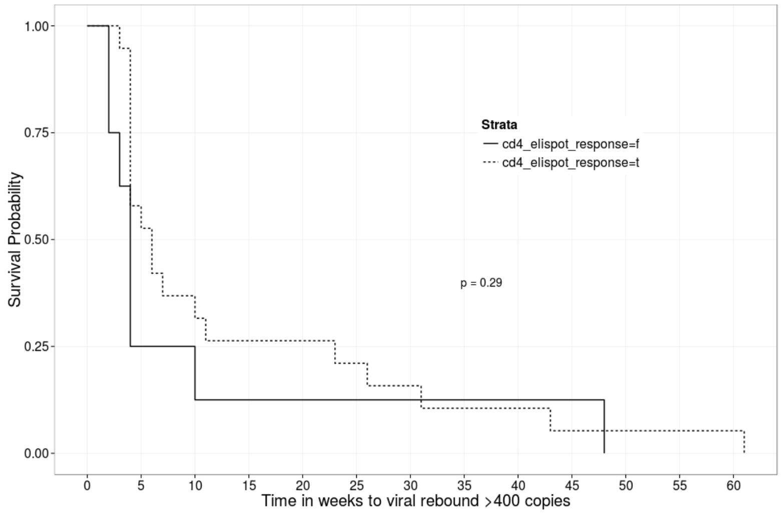

b.

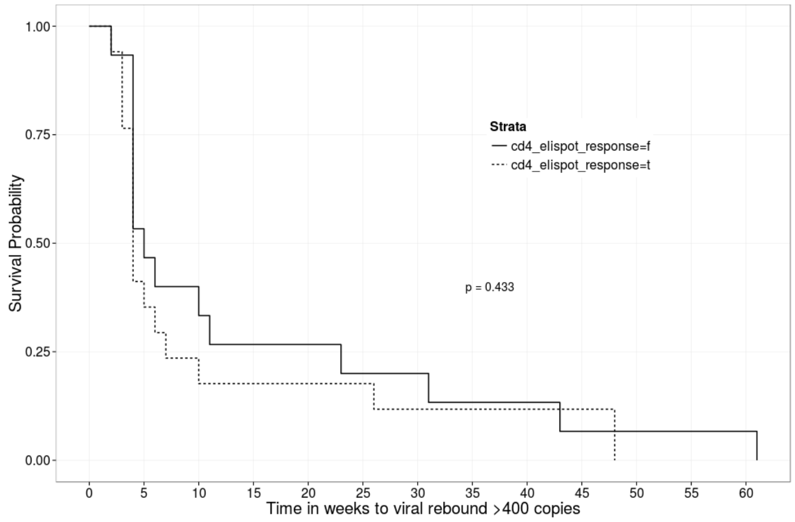

Supplementary Figure 1. Kaplan Meier analysis of time to viral rebound >400 copies/ml. Kaplan Meier analysis of time to viral rebound >400 copies/ml. Stratification is by response to p24 gag CD4+ ELISPOT at a) baseline and b) week 48. For both analyses the variable was stratified 'f' for false (no response) and 't' for true (response). (a) Stratification by baseline response to CD4+ ELISPOT (n=27). (b) Stratification by week 48 response to CD4+ ELISPOT (n=32)

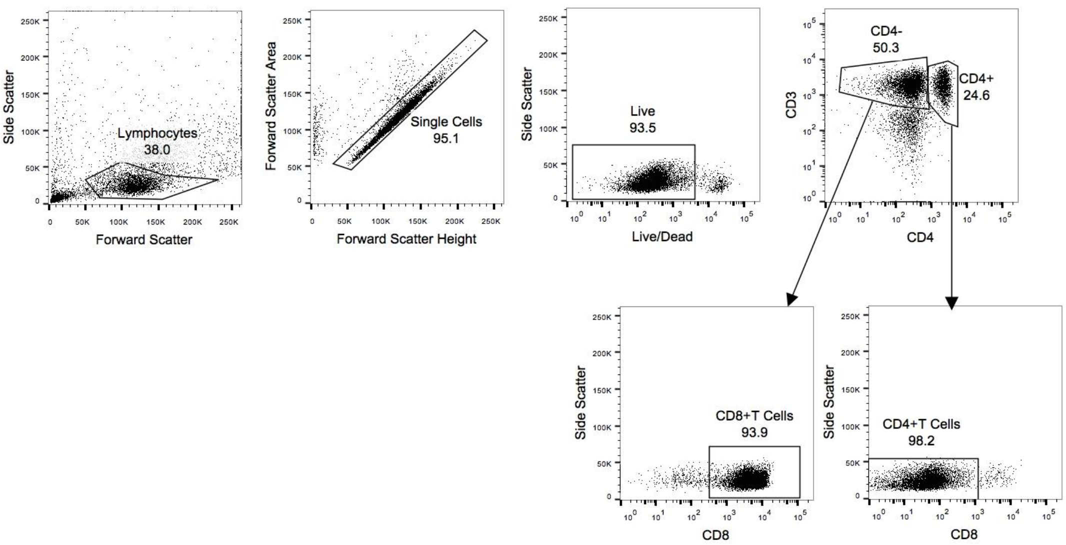

Supplementary Figure 2. Gating Strategy for FACS analysis. Thawed PBMC were first gated on forward and side scatter to identify lymphocytes after which singlets were gated followed by live cells. The remaining cells were then divided into CD4+T cells and CD8+T cells first on CD3 and CD4 expression followed by CD8 expression. Cells were then quantified according to expression of the exhaustion and activation markers described in Table S2.

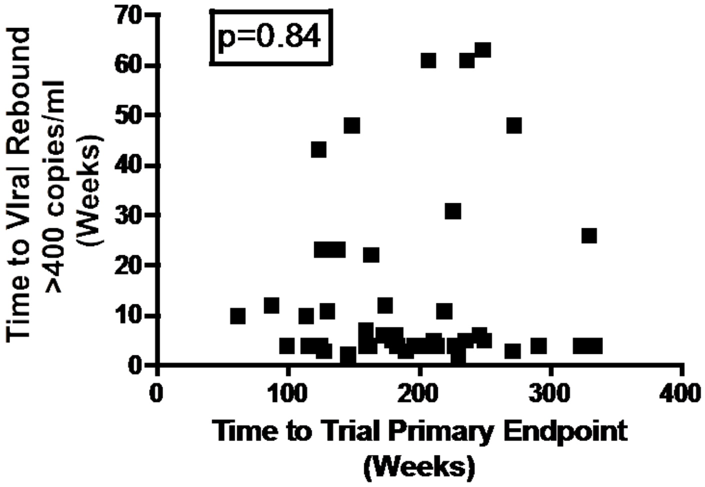

Supplementary Figure 3. No correlation between time to VL>400 and trial endpoint. Time to a viral load of >400 copies after ART cessation was compared with time to the trial primary endpoint in participants receiving 48 weeks of ART since PHI (n=46). Analysis is by Spearman's correlation. P=0.84.

Supplementary Table 1. Patient Demographics

	Total participants available for analysis pre-ART (week 0)	Participants at week 0 with full set of baseline immunology markers.	Participants available for analysis at TI after 48 weeks ART (week 48)
Number	154	78	47
Patients with a Total HIV-1 DNA test	154 (100%)	78 (100%)	47 (100%)
Patients with an Integrated HIV-1 test.	111 (72%)	61 (78%)	47 (100%)
Log10 baseline Total HIV-1 DNA copies/ml	3.88 (3.42- 4.24)	3.86 (3.46-4.19)	3.25 (2.90-3.44)
Log10 baseline Integrated HIV-1 DNA copies/ml	3.6 (3.26 – 3.79)	3.62 (3.33-3.83)	3.0 (2.7-3.2)
Time since seroconversion (days)	73.82 (49.2 – 95.8)	77 (58.75-97.51)	76.5 (52.0-96.9)
Log_{10} Viral Load copies/ml	4.62 (3.95 - 5.25)	4.62 (3.96-5.23)	UD*
CD4 cell count (cells/µl)	558 (428 – 680.9)	524 (405-655)	565 (439-732)
Country of recruitment	Australia 21 (13.6%)<br>Italy 18 (12%)<br>Brazil 13 (8.4%)<br>UK 102 (66.2%)	UK: 78 (100%)	Australia 5 (10.6%)<br>Italy 5 (10.6%)<br>Brazil 3 (6.4%)<br>UK 34 (72.3%)
Viral Subtype (%)	B (100%)	B (100%)	B (100%)
Sex	Female 4 (3%)<br>Male 150 (97%)	Female 0 (0%)<br>Male 78 (100%)	Female 1 (2.1%)<br>Male 46 (97.9%)

Details of participants included in the analyses undertaken at pre-therapy baseline (week 0) and at TI (week 48) for those individuals included in the ART-48 trial arm of SPARTAC. The middle column presents participants at week 0 who had measurements available for immunological biomarkers. Data shown are values (% of non-missing values) for categorical data or medians and interquartile ranges in brackets for continuous variables. *UD represents ‘undetectable’.

Supplementary Table 2. Patient Numbers Contributing to HLA Analyses in Figure 1c.

HLA A	n	HLA B	n	HLA C	n
A_0101	45	B_0702	32	C_0102	14
A_0201	75	B_0801	28	C_0202	18
A_0202	1	B_1302	5	C_0302	1
A_0205	2	B_1401	2	C_0303	15
A_0206	1	B_1402	8	C_0304	23
A_0213	1	B_1501	20	C_0401	31
A_0217	1	B_1502	1	C_0403	1
A_0238	1	B_1503	3	C_0501	21
A_0301	24	B_1507	1	C_0602	32
A_0302	1	B_1510	2	C_0701	38
A_0305	1	B_1517	2	C_0702	35
A_1101	17	B_1518	1	C_0704	6
A_2301	3	B_1537	1	C_0801	1
A_2402	29	B_1801	6	C_0802	10
A_2403	1	B_1803	2	C_1202	7
A_2405	1	B_2702	1	C_1203	16
A_2407	1	B_2705	15	C_1301	1
A_2601	10	B_3501	12	C_1402	1
A_2608	2	B_3502	3	C_1403	1
A_2901	4	B_3503	3	C_1502	3
A_2902	9	B_3504	1	C_1601	11
A_3001	4	B_3508	1	C_1602	3
A_3002	4	B_3521	1	C_1604	1
A_3004	2	B_3543	1	C_1701	2
A_3101	13	B_3701	10		
A_3201	9	B_3801	8		
A_3301	4	B_3802	1		
A_3601	1	B_3901/06/10/13	5/3/1/1		
A_6601	2	B_4001/02/10	15/1/1		
A_6801	8	B_4101	2		
A_6802	1	B_4402	23		
A_6901	1	B_4403	12		
A_7401	2	B_4405	4		
		B_4501	5		
		B_4901	2		
		B_5001	4		
		B_5101	10		
		B_5108	1		
		B_5201	7		
		B_5301	5		
		B_5501	5		
		B_5502	1		
		B_5701	11		
		B_5801	1		
		B_5802	1		

Details of the 4-digit HLA typing for participants included in Figure 1c. 2-digit numbers used for the analysis were summed from the 4-digit typing.

Supplementary Table 3. Impact of correcting for multiple testing using Bonferroni and False Positive Rates on the linear regression analysis

Explanatory Variable	Un-adjusted p-value	N=14, Bonferroni corrected	N=14 FDR corrected
CD8+ CD38	<0.001	<0.001	<0.001
CD4+ CD38	0.055	0.768	0.077
CD8 Lag-3	0.002	0.029	0.004
ddimer	<0.001	0.006	0.001
CD8 PD-1	0.002	0.029	0.004
CD4 PD-1	0.169	1	0.215
CD8 HLADR	0.005	0.067	0.008
CD4 HLADR	0.041	0.568	0.063
Viral RNA	<0.001	<0.001	<0.001
CD4 cell count	<0.001	<0.001	<0.001
CD4 ratio	<0.001	<0.001	<0.001

Adjusting the p-values from Table 1 for multiple comparisons for simple linear regressions with HIV-DNA. Adjustments are presented for 14 tests, the Bonferroni correction and false discovery rate (FDR) of Benjamini and Hochberg are presented.

Supplementary Table 4. Biomarkers at TI associated with Total HIV-1 DNA

Variable.	n	Simple linear model (β; se; p value)*
Log_{10} CA-RNA	27	0.297; 0.094; 0.004
CD4/CD8 ratio	36	-0.51; 0.455; 0.271
CD4 cell count per 100 cells	47	-0.035; 0.026; 0.176
CD8+ CD38+^	27	0.011; 0.02; 0.59
CD4+ CD38+^	27	-0.011; 0.022; 0.616
CD8+ PD-1+^	27	-0.096; 0.05; 0.089
CD8+ HLA-DR+^	27	-0.0021; 0.012; 0.859
CD4+ HLA-DR+^	27	-0.025; 0.006; < 0.001
CD4+PD-1^	27	0.0032; 0.011; 0.781
D-dimer	27	1.12; 0.636; 0.072

Linear regression model exploring associations with biomarkers and Total HIV-1 DNA measured at the point of TI (week 48). * β = regression coefficients, se = standard errors and p-values. TI: Treatment interruption; CA-RNA: unspliced cell-associated HIV-1 RNA. ^: expression on T cells

Supplementary Table 5. Cox Proportional hazard models predicting viral rebound (>400 copies/ml) adjusting for one other exhaustion marker measured at baseline.

**Biomarker Expression on T cells**<br>**N=20**	**HR (CI) p-value**
**PD-1+ CD4+**<br>**Tim3+ CD4+**	**1.24 (0.99-1.55) p=0.056**<br>**1.25 (1.09-1.41) p<0.001**
**PD-1+ CD8+**<br>**Tim3+ CD8+**	**1.18 (1.024-1.365) p=0.022**<br>**1.11 (1.039-1.205) p=0.00301**
**PD-1+ CD4+**<br>**Lag-3+ CD4+**	**1.19 (0.91-1.57) p=0.19**<br>**1.05 (0.989-1.131) p=0.096**
**PD-1+ CD8+**<br>**Lag3+ CD8+**	**1.16 (1.005-1.348) p=0.043**<br>**1.1 (1.02-1.19) p=0.013**
**Tim-3+ CD4+**<br>**Lag-3+ CD4+**	**1.24 (1.09-1.4) p<0.001**<br>**1.05 (0.99-1.11) p=0.08**
**Tim-3+ CD8+**<br>**Lag-3+ CD8+**	**1.17 (1.063-1.28) p=0.0012**<br>**1.14 (1.05-1.24) p=0.0019**

Supplementary Table 6. Impact of correcting for multiple testing using Bonferroni and False Positive Rates on the Kaplan-Meier Survival analysis

Kaplan-Meier tests	Unadjusted P-values	P-value Bonferroni corrected	P-value false discovery rate corrected
CD4+ PD-1	0.00017	0.0029	0.0029
CD4+ Tim-3	0.0032	0.055	0.0183
CD4+ Lag-3	0.016	0.276	0.069
CD8+ PD-1	0.0013	0.0223	0.0111
CD8+ Tim-3	0.08	1.0	0.226
CD8+ Lag-3	0.023	0.39	0.079

The Kaplan-Meir p-values from Figure 3 were adjusted for 17 tests. Two methods are presented: the more conservative Bonferroni method and the false discovery rate of Benjamini and Hochberg.

Reference for the FDR method: Benjamini, Y., and Hochberg, Y. (1995). Controlling the false discovery rate: a practical and powerful approach to multiple testing. Journal of the Royal Statistical Society Series B 57, 289–300.

Supplementary Table 7. Univariable Cox Proportional hazard models predicting viral rebound >50 copies/ml after TI for biomarkers measured at baseline

Biomarker Expression on T cells N=20	HR (CI) p-value
	Rebound to 50 RNA/copies/ml
PD-1 CD4+	1.29 (1.036 – 1.6) p=0.023
PD-1 CD8+	1.09 (0.78-1.07) p=0.214
Lag-3 CD4+	1.04 (0.98-1.1) p=0.128
Lag-3 CD8+	1.001 (0.97-1.027) p=0.081
Tim-3 CD4+	1.21 (1.09-1.35) p<0.001
Tim-3 CD8+	1.108 (1.029-1.19) p=0.0067

Supplementary Table 8. Univariable Cox Proportional hazard models predicting viral rebound >400 copies/ml for biomarkers measured at TI

Biomarker Expression on T cells N=39	HR (CI) p-value
	Rebound to 400 RNA/copies/ml
PD-1 CD4+	0.99 (0.93 – 1.05) p=0.82
PD-1 CD8+	0.92 (0.78-1.07) p=0.303
Lag-3 CD4+	1.04 (0.96-1.13) p=0.322
Lag-3 CD8+	1.001 (0.97-1.027) p=0.92
Tim-3 CD4+	1.02 (0.96-1.083) p=0.466
Tim-3 CD8+	1.027 (0.85-1.239) p=0.783

Supplementary Note 1

We thank the participants of SPARTAC and the SPARTAC Trial Investigators: Trial Steering Committee: Independent Members- A Breckenridge (Chair), P Clayden, C Conlon, F Conradie, J Kaldor*, F Maggiolo, F Ssali, Country Principal Investigators - P Kaleebu, G Ramjee, JM Miro. Data and Safety Monitoring Committee (DSMC): T Peto (Chair), A McLaren (in memoriam), V Beral, G Chene, J Hakim. Co-ordinating Trial Centre: Medical Research Council Clinical Trials Unit, London (K Porter, M Thomason, F Ewings, M Gabriel, D Johnson, K Thompson, A Cursley*, K Donegan*, E Fossey*, P Kelleher*, K Lee*, B Murphy*, D Nock*). Central Immunology Laboratories and Repositories: The Peter Medawar Building for Pathogen Research, University of Oxford, UK (L Ohm Laursen*, P Goulder). Central Virology Laboratories and Repositories: Jefferiss Trust Laboratories, Imperial College, London, UK (M McClure, D Bonsall*, O Erlwein*, A Helander*, S Kaye, M Robinson, L Cook*, G Adcock*, P Ahmed*). Clinical Endpoint Review Committee: N Paton,. Investigators and Staff at Participating Sites: Australia: St Vincents Hospital, Sydney, Northside Clinic, Melbourne (R Moore), East Sydney Doctors, Sydney (R McFarlane), Prahran Market Clinic, Melbourne (N Roth), Taylor Square Private Clinic, Sydney (R Finlayson), The Centre Clinic, Melbourne (B Kiem Tee), Sexual Health Centre, Melbourne (T Read), AIDS Medical Unit, Brisbane (M Kelly), Burwood Rd Practice, Sydney (N Doong), Holdsworth House Medical Practice, Sydney (M Bloch), Aids Research Initiative, Sydney (C Workman). Coordinating Centre in Australia: Kirby Institute University of New South Wales, Sydney (P Grey, M Law). Brazil: Projeto Praca Onze, Hospital Escola Sao Francisco de Assis, Universidade federal do Rio de Janeiro, Rio de Janeiro (P Gama, M Mercon*, M Barbosa de Souza, C Beppu Yoshida, JR Grangeiro da Silva, A Sampaio Amaral, D Fernandes de Aguiar, M de Fatima Melo, R Quaresma Garrido). Italy: Ospedale San Raffaele, Milan (S Nozza, M Pogliaghi, S Chiappetta, L Della Torre, E Gasparotto), Ospedale Lazzaro Spallanzani, Roma (G DOffizi, C Vlassi, A Corpolongo). South Africa: Cape Town: Desmond Tutu HIV-1 Centre, Institute of Infectious Diseases, Cape Town (R Wood, J Pitt, C Orrell, F Cilliers, R Croxford, K Middelkoop, LG Bekker, C Heiberg, J Aploon, N Killa, E Fielder, T Buhler). Johannesburg: The Wits Reproductive Health and HIV-1 Institute, University of Witswatersrand, Hillbrow Health Precinct, Johannesburg (H Rees, F Venter, T Palanee), Contract Laboratory

Services, Johannesburg Hospital, Johannesburg (W Stevens, C Ingram, M Majam, M Papathanasopoulos). Kwazulu-Natal: HIV-1 Prevention Unit, Medical Research Council, Durban (G Ramjee, S Gappoo, J Moodley, A Premrajh, L Zako). Uganda: Medical Research Council/Uganda Virus Research Institute, Entebbe (H Grosskurth, A Kamali, P Kaleebu, U Bahemuka, J Mugisha*, HF Njaj*). Spain: Hospital Clinic-IDIBAPS, University of Barcelona, Barcelona (JM Miro, M Lopez-Dieguez*, C Manzardo, JA Arnaiz, T Pumarola, M Plana, M Tuset, MC Ligero, MT Garca, T Gallart, JM Gatell). UK and Ireland: Royal Sussex County Hospital, Brighton (K Hobbs, N Perry, D Pao, D Maitland, L Heald), St James's Hospital, Dublin (F Mulcahy, G Courtney, S O'Dea, D Reidy), Regional Infectious Diseases Unit, Western General Hospital and Genitourinary Dept, Royal Infirmary of Edinburgh, Edinburgh (C Leen, G Scott, L Ellis, S Morris, P Simmonds), Chelsea and Westminster Hospital, London (B Gazzard, D Hawkins, C Higgs), Homerton Hospital, London (J Anderson, S Mguni), Mortimer Market Centre, London (I Williams, N De Esteban, P Pellegrino, A Arenas-Pinto, D Cornforth*, J Turner*), North Middlesex Hospital (J Ainsworth, A Waters), Royal Free Hospital, London (M Johnson, A Carroll, P Byrne, Z Cuthbertson), Barts & the London NHS Trust, London (C Orkin, J Hand, C De Souza), St Marys Hospital, London (E Thomson*, J Fox*, K Legg, S Mullaney*, A Winston, S Wilson, P Ambrose), Birmingham Heartlands Hospital, Birmingham (S Taylor, G Gilleran). Imperial College Trial Secretariat: S Keeling, A Becker. Imperial College DSMC Secretariat: C Boocock.

* Left the study team before the trial ended.
